# Supplementary material for: Structural Characterization of a Pleurotus sajor-caju Polysaccharide and Its Neuroprotection Related to the Inhibition of Oxidative Stress
Source: Nutrients. 2022 Sep 29;14(19):4047. doi: 10.3390/nu14194047 (PMC9573675; doi:10.3390/nu14194047)
Supplement: Supplementary file 1 [file nutrients-14-04047-s001.zip › nutrients-1922894-supplementary.pdf]

nutrients

MDPI

Supplementary data

Structural Characterization of *Pleurotus sajor-caju* polysaccharides and its neuroprotection related to inhibition on oxidative stress

Mengdi Liu [1,2,†], Yingdi Dai [1,2,†], Chengming Song [2], Jia Wang [3], Yang Liu [1,2*] and Qi Wang [1,2*]

[1] Engineering Research Center of Chinese Ministry of Education for Edible and Medicinal Fungi, Jilin Agricultural University, Changchun 130118, China

[2] College of Plant Protection, Jilin Agricultural University, Changchun 130118, China

[3] Guang'anmen Hospital China Academy of Chinese Medical Sciences Respiratory Department, Beijing 100053, China

* Correspondence: y_liu10@jlau.edu.cn (Y.L.); qiwang@jlau.edu.cn (Q.W.); Tel./Fax: +86-431-84532989(Y.L.); Tel./Fax: +86-431-84533269 (Q.W.)

† These authors contributed equally to this work.

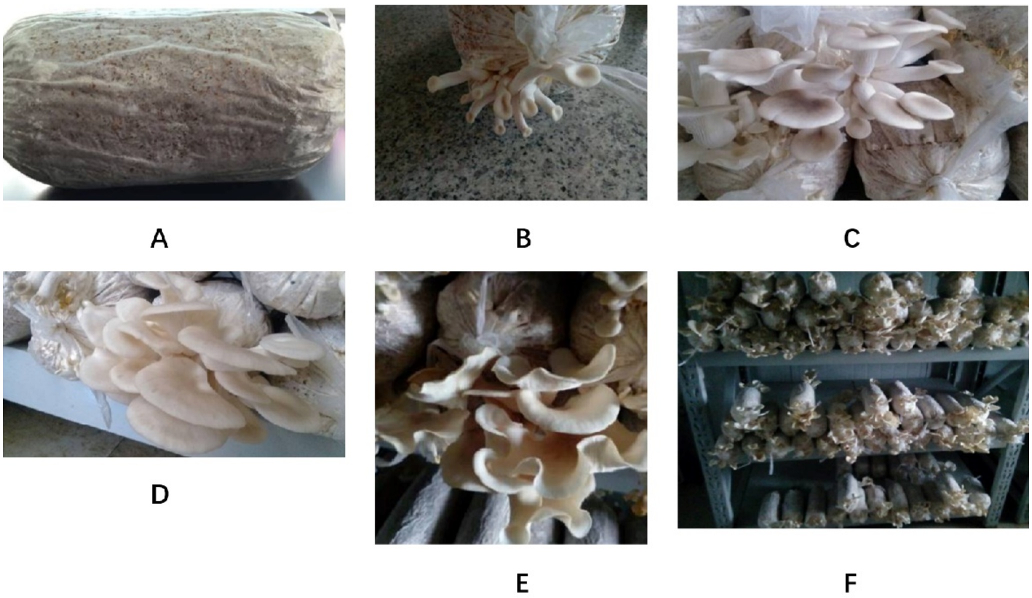

Figure S1. Results of PCR for ITS sequences. (**A**) Primordium formation stage. (**B**) Mulberry period. (**C**) Coral period. (**D**) Growing period. (**E**) The mature period. (**F**) Indoor growing environment.

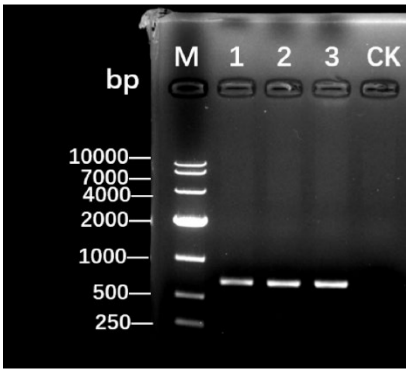

Figure S2. Results of PCR for ITS sequences

M: DNA maker, CK: negative control. 1-3 was three parallel experiments of the sample.

```
Sequence ID: Query_33249  Length: 694  Number of Matches: 1

Range 1: 1 to 659  Graphics                                   ▼ Next Match ▲

Score              Expect   Identities        Gaps          Strand
1197 bits(648)     0.0      656/659(99%)      3/659(0%)     Plus/Minus

Query  11   AGT-CTA-CTGATTTGAGGTCAA-TTGTCAAATTGTCCTTGCGGACGATTAGAGAGCTGG  67
            ||| ||| ||||||||||||||| ||||||||||||||||||||||||||||||||||||
Sbjct  659  AGTCCTACCTGATTTGAGGTCAAATTGTCAAATTGTCCTTGCGGACGATTAGAGAGCTGG  600

Query  68   ACTCTATTCATGCGTGCTATTGATGAGTGATAATTATCACATCATGCGCAGAGGCAATGA  127
            ||||||||||||||||||||||||||||||||||||||||||||||||||||||||||||
Sbjct  599  ACTCTATTCATGCGTGCTATTGATGAGTGATAATTATCACATCATGCGCAGAGGCAATGA  540

Query  128  GAAGTCCTGCTAATGCATTTAAGAGGAGCCGACTTGTTACAGCCAGCAACCCCCAACAAT  187
            ||||||||||||||||||||||||||||||||||||||||||||||||||||||||||||
Sbjct  539  GAAGTCCTGCTAATGCATTTAAGAGGAGCCGACTTGTTACAGCCAGCAACCCCCAACAAT  480

Query  188  CCAAACATCACAAATAAATGTGAGTTTGAGAATTTAATGACACTCAAACAGGCATGCCCC  247
            ||||||||||||||||||||||||||||||||||||||||||||||||||||||||||||
Sbjct  479  CCAAACATCACAAATAAATGTGAGTTTGAGAATTTAATGACACTCAAACAGGCATGCCCC  420

Query  248  TCGGAATACCAAGGGGCGCAAGGTGCGTTCAAAGATTCGATGATTCACTGAATTCTGCAA  307
            ||||||||||||||||||||||||||||||||||||||||||||||||||||||||||||
Sbjct  419  TCGGAATACCAAGGGGCGCAAGGTGCGTTCAAAGATTCGATGATTCACTGAATTCTGCAA  360

Query  308  TTCACATTACTTATCGCATTTCGCTGCGTTCTTCATCGATGCGAGAGCCAAGAGATCCGT  367
            ||||||||||||||||||||||||||||||||||||||||||||||||||||||||||||
Sbjct  359  TTCACATTACTTATCGCATTTCGCTGCGTTCTTCATCGATGCGAGAGCCAAGAGATCCGT  300

Query  368  TGTTGAAAGTTGTATTATGGTTTATAGGCACAAGGCCCATTAAATGACATTCGTAGACAT  427
            ||||||||||||||||||||||||||||||||||||||||||||||||||||||||||||
Sbjct  299  TGTTGAAAGTTGTATTATGGTTTATAGGCACAAGGCCCATTAAATGACATTCGTAGACAT  240

Query  428  ACATTTGGGGTGTGTTAAGTAAATAGACTGCGTTGTCACACCGAGACGTTTAAATCCCAG  487
            ||||||||||||||||||||||||||||||||||||||||||||||||||||||||||||
Sbjct  239  ACATTTGGGGTGTGTTAAGTAAATAGACTGCGTTGTCACACCGAGACGTTTAAATCCCAG  180

Query  488  CAAACCAAGTCTGACGACTTGAAGGACGACTTCACAGATCTATCAAAAGTTCACAGGTGG  547
            ||||||||||||||||||||||||||||||||||||||||||||||||||||||||||||
Sbjct  179  CAAACCAAGTCTGACGACTTGAAGGACGACTTCACAGATCTATCAAAAGTTCACAGGTGG  120

Query  548  TTGAAAGACTAGTGAAGCGTGCACATGCCCCTAGAGGCCAGCAACAACTCCATAGTGAAT  607
            ||||||||||||||||||||||||||||||||||||||||||||||||||||||||||||
Sbjct  119  TTGAAAGACTAGTGAAGCGTGCACATGCCCCTAGAGGCCAGCAACAACTCCATAGTGAAT  60

Query  608  TCATTAATGATCCTTCCGCAGGTTCACCTACGGAAACCTTGTTACGACTTTTACTTCCA  666
            |||||||||||||||||||||||||||||||||||||||||||||||||||||||||||
Sbjct  59   TCATTAATGATCCTTCCGCAGGTTCACCTACGGAAACCTTGTTACGACTTTTACTTCCA  1
```

Figure S3. Results of ITS sequence alignment (Compared with *Pleurotus sajor-caju* GenBank: AY540328.1 on NCBI, the similarity was over 99%, and it was identified as *Pleurotus sajor-caju*.)
